# Nrf2 activation by pyrroloquinoline quinone inhibits natural aging‐related intervertebral disk degeneration in mice

**DOI:** 10.1111/acel.14202

**Published:** 2024-05-23

**Authors:** Qi Xue, Jie Li, Ran Qin, Mingying Li, Yiping Li, Jing Zhang, Rong Wang, David Goltzman, Dengshun Miao, Renlei Yang

**Affiliations:** ^1^ Department of Plastic Surgery Affiliated Friendship Plastic Surgery Hospital of Nanjing Medical University, Nanjing Medical University Nanjing China; ^2^ The Research Center for Bone and Stem Cells, Department of Anatomy, Histology and Embryology Nanjing Medical University Nanjing China; ^3^ Department of Orthopaedics Nanjing First Hospital Nanjing China; ^4^ Shenzhen Key Laboratory for Systemic Aging and Intervention Shenzhen University Shenzhen China; ^5^ Calcium Research Laboratory, McGill University Health Centre and Department of Medicine McGill University Montreal Quebec Canada

**Keywords:** aging, IVDD, Keap1–Nrf2 signaling, pyrroloquinoline quinone, Wnt5a

## Abstract

Age‐related intervertebral disk degeneration (IVDD) involves increased oxidative damage, cellular senescence, and matrix degradation. Pyrroloquinoline quinone (PQQ) is a water‐soluble vitamin‐like compound with strong anti‐oxidant capacity. The goal of this study was to determine whether PQQ can prevent aging‐related IVDD, and the underlying mechanism. Here, we found that dietary PQQ supplementation for 12 months alleviated IVDD phenotypes in aged mice, including increased disk height index and reduced histological scores and cell loss, without toxicity. Mechanistically, PQQ inhibited oxidative stress, cellular senescence, and senescence‐associated secretory phenotype (SASP) in the nucleus pulposus and annulus fibrosus of aged mice. Similarly, PQQ protected against interleukin‐1β‐induced matrix degradation, reactive oxygen species accumulation, and senescence in human nucleus pulposus cells (NPCs) in vitro. Molecular docking predicted and biochemical assays validated that PQQ interacts with specific residues to dissociate the Keap1–Nrf2 complex, thereby increasing nuclear Nrf2 translocation and activation of Nrf2‐ARE signaling. RNA sequencing and luciferase assays revealed Nrf2 can transcriptionally upregulate Wnt5a by binding to its promoter, while Wnt5a knockdown prevented PQQ inhibition of matrix metalloproteinase‐13 in NPCs. Notably, PQQ supplementation failed to alleviate aging‐associated IVDD phenotypes and oxidative stress in aged Nrf2 knockout mice, indicating Nrf2 is indispensable for PQQ bioactivities. Collectively, this study demonstrates Nrf2 activation by PQQ inhibits aging‐induced IVDD by attenuating cellular senescence and matrix degradation. This study clarifies Keap1–Nrf2–Wnt5a axis as the novel signaling underlying the protective effects of PQQ against aging‐related IVDD, and provides evidence for PQQ as a potential agent for clinical prevention and treatment of natural aging‐induced IVDD.

## Graphical abstract

This study sheds light on the preventive effects of dietary PQQ supplementation on oxidative stress, cellular senescence, and extracellular matrix (ECM) degradation in nucleus pulposus cells (NPCs) through Keap1–Nrf2 signaling‐mediated stress response and Wnt5a upregulation, thus preventing aging‐related IVDD.

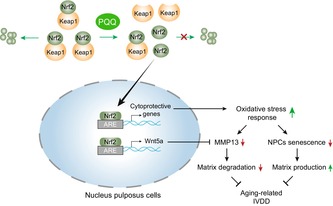

**Abbreviations**
ALT: alanine aminotransferase
ARE: antioxidant response elements
AST: aminotransferase
ChIP‐qPCR: Chromatin immunoprecipitation‐quantitative PCR
Co‐IP: co‐immunoprecipitation
Collagen II: collagen type II
DEGs: Differentially expressed genes
DHE: dihydroethidium
DHI: disc height index
ECM: extracellular matrix
EDTA: ethylene diamine tetraacetic acid
EdU: 5‐ethynyl‐2‐deoxyuridine
FDA: Food and Drug Administration
HO1: heme oxygenase1
IVDD: intervertebral disc degeneration
Keap1: kelch‐like ECH‐associated protein 1
Mmp13: matrix metalloproteinase 13
NPCs: nucleus pulposus cells
Nrf2: nuclear factor erythroid 2‐related factor 2
PDB: Protein Data Bank
PQQ: pyrroloquinoline quinone
ROS: reactive oxygen species
SASP: senescence‐associated secretory phenotype
SDF: Structure Data File
SO&FG: Safranin O and fast green
tBHQ: tert‐butylhydroquinone
Wnt5a: wingless‐type MMTV integration site family, member 5A

## 1 INTRODUCTION

Aging stands as a pivotal risk factor for numerous chronic conditions, prominently including intervertebral disk degeneration (IVDD). Cellular senescence, a fundamental aging mechanism implicated in multiple age‐related disorders, plays a significant role in driving IVDD as well. Previous studies have underscored the accumulation of senescent cells in degenerate intervertebral disks with aging (Le Maitre et al., 2007; Vo et al., 2010), emphasizing the potential of targeting senescent cells and their senescence‐associated secretory phenotype (SASP) for IVDD prevention (Novais et al., 2019, 2021; Patil et al., 2019). The accumulation of reactive oxygen species (ROS), acknowledged as a key driver of cellular senescence and aging, intensifies with aging and plays a pivotal role in IVDD pathogenesis (López‐Otín et al., 2023). Notably, degenerated intervertebral disks exhibit heightened oxidative stress and oxidation production, further contributing to the progression of IVDD. Therapies targeting oxidative stress have shown promise in alleviating or preventing IVDD progression (Ma et al., 2022; Xu et al., 2019).

Pyrroloquinoline quinone (PQQ), initially identified as a cofactor for bacterial oxidoreductase, has demonstrated systemic effects in mice, including growth failure, immune dysfunction, and impaired reproductive capacity when deficient in foods (Killgore et al., 1989; Steinberg et al., 1994). Conversely, PQQ supplementation has shown efficacy in mitigating the progression of various diseases in mice, owing largely to its antioxidant function (Dai et al., 2022; Jonscher et al., 2017; Ma et al., 2019), with radical‐scavenging activity reported to be 7.4‐fold higher than that of vitamin C (Akagawa et al., 2016). Although PQQ cannot be synthesized in mammals, it is present in foods such as meat, milk, and vegetables (Mitchell et al., 1999; Noji et al., 2007), rendering it an attractive option for addressing oxidative stress‐induced diseases, including IVDD. Previous studies from our group have indicated that PQQ supplementation can alleviate several diseases, including estrogen deficiency‐ and aging‐related osteoporosis in mice (Li et al., 2023); its potential in alleviating natural aging‐related IVDD remains unexplored.

Nuclear factor erythroid 2‐related factor 2 (Nrf2) is a widely expressed transcription factor pivotal in regulating the expression of antioxidant‐related genes to maintain cellular redox homeostasis (Niture et al., 2014). Under basal conditions, Nrf2 binds to its inhibitor Kelch‐like ECH‐associated protein 1 (Keap1), facilitating the degradation of Nrf2 via the 26S‐proteasome (Cullinan et al., 2004). Conversely, upon electrophilic/oxidative stress, Keap1 activity is inhibited, resulting in the release of Nrf2, which translocates to the nucleus and activates antioxidant response elements (AREs) on genes encoding antioxidant and detoxification enzymes (Suzuki & Yamamoto, 2015; Tonelli et al., 2018). Numerous proteins and compounds have been identified to disrupt the Keap1–Nrf2 complex, promoting Nrf2‐mediated cellular redox homeostasis (Ge et al., 2017; Komatsu et al., 2010; Mulvaney et al., 2016). Recent evidence has highlighted the pivotal role of the Nrf2 signaling pathway in preventing against surgery‐induced IVDD (Ma et al., 2022; Tang et al., 2019). However, the impact of Nrf2 deficiency or activation on natural aging‐induced IVDD in mice remains unclear.

This study aims to investigate the role and mechanism of dietary PQQ supplementation in preventing natural age‐related IVDD. We demonstrate that prolonged PQQ supplementation attenuates age‐associated increases in oxidative stress accumulation, cellular senescence, and extracellular matrix (ECM) degradation. The findings shed light on the mechanism through which PQQ inhibits oxidative stress and prevents age‐related IVDD via Keap1–Nrf2 signaling activation, positioning PQQ as an ideal agent for the prevention and treatment of natural aging‐induced IVDD.

## 2 MATERIALS AND METHODS

### 2.1 Animal experiments

Twenty‐four 12‐month‐old C57BL/6 male wild‐type mice were randomly divided into three groups. Control groups comprised young (12‐month‐old) and aging (24‐month‐old) wild‐type mice on a normal diet, while the experimental group received a PQQ‐containing diet (4 mg/kg diet) from 12 months of age for 12 months. Nrf2^+/−^ mice were obtained from Cyagen Biosciences Inc. To investigate the role of Nrf2 in the protective effect of PQQ on IVDD, 6‐month‐old wild‐type and Nrf2^−/−^ mice were given either a normal or PQQ‐containing diet for 12 months. All animal experiments adhered to the guidelines approved by the Institutional Animal Care and Use Committee of Nanjing Medical University.

### 2.2 X‐ray and data analysis

After sacrifice, X‐ray images were taken, and the disk height index (DHI) was calculated by averaging values from the posterior, middle, and anterior parts of the disk and dividing by the average height of the adjacent vertebral body.

### 2.3 Histology and immunohistochemical examination

Slides of each disk were stained with Safranin O and Fast green (SO). Histopathological analysis was evaluated by histological grading system, using a microscope (ZEISS). The histologic score was 5 for normal disk, 6–10 for moderately degenerated disk, and 11–15 for severely degenerated disk as previously described (Tam et al., 2018). Immunohistochemical staining was performed as previously described (Chen et al., 2023). Primary antibodies against rabbit anti‐Mmp13 (Proteintech, 16396‐1‐AP), rabbit anti‐COL2A1 (Bioss, bs‐10589R), rabbit anti‐p16 (Abcam, ab211542) rabbit anti‐Nrf2 (Proteintech, 16396‐1‐AP), Mouse anti‐8‐OHdG (Abcam, ab62623), rabbit anti‐IL‐6 (Santa Cruz, sc‐1265), rabbit anti‐IL‐1β (Abcam, ab9722) were used. For 5‐ethynyl‐2‐deoxyuridine (EdU) assay to evaluate cell proliferation, cultured human NPCs were incubated with EdU for 2 h, after which EdU staining was performed using Apollo567 Stain Kit (RiboBio). Senescent cells were detected using a senescence β‐Galactosidase staining kit (Beyotime) according to the manufacturer's instructions. Cell apoptosis was assessed using a TUNEL kit (Beyotime, C1091) according to the manufacturer's guidelines.

### 2.4 Cell cultures and treatments

Human NPCs were kindly provided by Prof. Yongxin Ren. Primary NPCs isolated from mice were described previously (Wang, Yang, et al., 2023). Briefly, the gel‐like NP was isolated from the anulus fibrosus under a microscope and treated with 0.1% type II collagenase (Beyotime, China) for 2 h at 37°C to digest the tissue completely. After centrifugation at 1500 rpm for 5 min, the precipitates were resuspended and placed in high glucose DMEM (Gibco) medium containing 10% FBS and 1% penicillin/streptomycin antibiotics in an incubator maintained with 5% CO_2_ at 37°C. When confluent, the NP cells were harvested using trypsin–EDTA solution (Gibco) and subcultured into suitable petri dish for following experiments. In vitro, human NPCs were pretreated with either vehicle or 10 μM PQQ (dissolved in ddH_2_O) for 12 h, and then, the cells were exposed to IL‐1β (10 ng/mL) in the presence of 10 μM PQQ. Subsequently, the cells were utilized for DHE staining and EdU labeling 24 h later, and for CCK8 assay and SA‐β‐gal staining 7 days later.

### 2.5 RNA‐seq and data analysis

Total RNA was extracted using TRIzol Reagent/RNeasy Mini Kit (Qiagen) according to the manufacturer's protocol. Quality control, sequencing, and data analysis were done by GENEWIZ (Suzhou, China). Briefly, 1 μg of total RNA was used to construct sequencing libraries using R8. VAHTS mRNA‐seq V3 Library Prep Kit for Illumina. Then, libraries were multiplexed and loaded on an Illumina Novaseq 6000 platform. Low quality reads were trimmed and paired clean reads were then aligned to the mm10 reference genome via software Hisat2 (v2.0.1). Reads were then counted using HTSeq (v0.6.1). Differentially expressed genes (DEGs) were calculated using DESeq2, and *p*adj (adjusted *p* value) <0.05 was used to detect DEGs.

### 2.6 Molecular modeling

The SDF (Structure Data File) file of PQQ was obtained from PubChem (https://pubchem.ncbi.nlm.nih.gov/compoundcompound/1024) and the effective targets were predicted using the PharmMapper Server (http://www.lilab‐ecust.cn/pharmmapper/) by employing the “All Targets” model. Autodock Vina 1.1.2 was employed to investigate the binding affinity and binding sites between PQQ and Nrf2–Keap1 complex. Briefly, the SDF file of PQQ was converted into PDBQT format by using AutoDock tools. The 3D crystal structure of the Nrf2–Keap1 complex was obtained from the Uniprot (http://www.uniprot.org, PDB ID: AF‐P25206‐F1). The docking protocol was generated as described previously (Li et al., 2023), and the partial diagram of molecular docking was generated using the PyMol software.

### 2.7 Plasmid construction

His‐Nrf2 expression plasmid was purchased from PPL (Public Protein/Plasmid Library, China). The indicated shRNAs targeting Wnt5a and Nrf2 were cloned to the lentivirus shRNA vector as previously described (Yang et al., 2022). For ARE‐driven luciferase reporter assay, a total of eight ARE copies were cloned to a pGL3‐promoter as we previously described (Yang et al., 2022). All constructs were verified by DNA sequencing (Tsingke, Nanjing).

### 2.8 Lentivirus production

Lentivirus particles were generated from HEK293T cells as previously described (Yang et al., 2022). Briefly, HEK293T cells were co‐transfected with transfer plasmid and packaging plasmids by Lipofectamine 2000 transfection reagent (Invitrogen). Viral particle‐containing supernatants were harvested 24 and 48 h later and were concentrated by ultra‐centrifugation (4000 × *g* for 3 h).

### 2.9 Co‐immunoprecipitation

For endogenous co‐immunoprecipitation (Co‐IP), NPCs were lysed in IP lysis buffer (20 mM Tris [pH 7.8], 150 mM NaCl, 0.2% NP‐40, 10% glycerol, supplemented with protease inhibitors cocktail [Roche Complete]) for 15 min, after which the cleared lysates were collected by centrifugation and incubated with the indicated antibody or control IgG coupled to Protein A/G agarose beads (Invitrogen, USA) at 4°C overnight under rotation. After being washed three times with IP lysis buffer, the immunoprecipitants were eluted by boiling in Laemmli loading buffer and analyzed using immunoblotting.

### 2.10 Detection of ROS

Reactive oxygen species evaluation of living cells in vitro using Dihydroethidium (DHE, ApexBio) was performed as previously described (Li et al., 2023). The fluorescence was captured under a microscope, and the intracellular intensity of DHE was quantified using Image J.

### 2.11 ChIP‐qPCR

ChIP‐qPCR was performed using the ChIP kit (Millipore) as previously described (Yang et al., 2020). The antibodies used in ChIP‐qPCR were as follows: rabbit anti‐IgG (Abcam, ab172730) and rabbit anti‐Nrf2 (Proteintech). The enriched DNA was used for qPCR to detect the putative ARE of Wnt5. The sequences of primers were listed in Table S1.

### 2.12 Dual luciferase assay

The indicated length of the Wnt5 promoter and its mutant were directly synthesized and cloned to pGL3‐basic (Promega). For ARE‐driven luciferase reporter assay, NPCs were co‐transfected with ARE‐driven luciferase, pcDNA3.1‐Nrf2, and renilla followed without or with PQQ treatment. Forty‐eight hours later, relative luciferase activity was measured using a kit (Promega) according to the manufacturer's instructions. The promoter sequences of Wnt5 and its mutant sequence cloned to pGL3‐basic are listed in Table S3.

### 2.13 Western blot

Whole‐cell lysates extraction and immunoblotting were performed as previously described (Yang et al., 2020). Primary antibodies against rabbit anti‐Nrf2 (Proteintech, 16396‐1‐AP), mouse anti‐p16 (Santa Cruz, sc‐1661), mouse anti‐p21 (Santa Cruz, sc‐6246), rabbit anti‐ Lamin B1 (Abcam, ab16048), rabbit anti‐HO‐1 (Proteintech, 10701‐1‐AP), rabbit anti‐COL2A1 (Bioss, bs‐10589R), rabbit anti‐Wnt5a (Proteintech, 55184‐1‐AP), rabbit anti‐Mmp13 (Proteintech, 16396‐1‐AP), rabbit anti‐Keap1 (Proteintech, 10503‐2‐AP), rabbit anti‐ubiquitin (Cell Signaling Technology, 43124), mouse anti‐GAPDH (Beyotime, AF0006), and rabbit anti‐Histone‐H3 (Proteintech, 17168‐1‐AP) were used in this study. GAPDH was used to normalize changes in specific gene expressions detected using Western blots.

### 2.14 RNA isolation and real‐time RT‐PCR

Total RNA extraction, cDNA synthesis, and real‐time RT‐PCR were performed as previously described (Yang et al., 2022). Gapdh was amplified at the same time to normalize gene expression. The PCR primer sequences used in this study are shown in Table S2.

### 2.15 Statistical analysis

Measured data were described as mean ± SD. The statistical analyses were performed using GraphPad Prism (Version 8.0). Two‐tailed Student's *t* test was used to compare differences between two groups in Figure 5h and Figures S5A and S7B. For multiple comparisons, one‐way ANOVA analysis of variance followed by Tukey's post hoc test was used. *p* values <0.05, <0.01, and <0.001 were considered statistically significant (*, **, ***).

## 3 RESULTS

### 3.1 Prevention of natural aging‐related IVDD by PQQ supplementation

**FIGURE 1:**
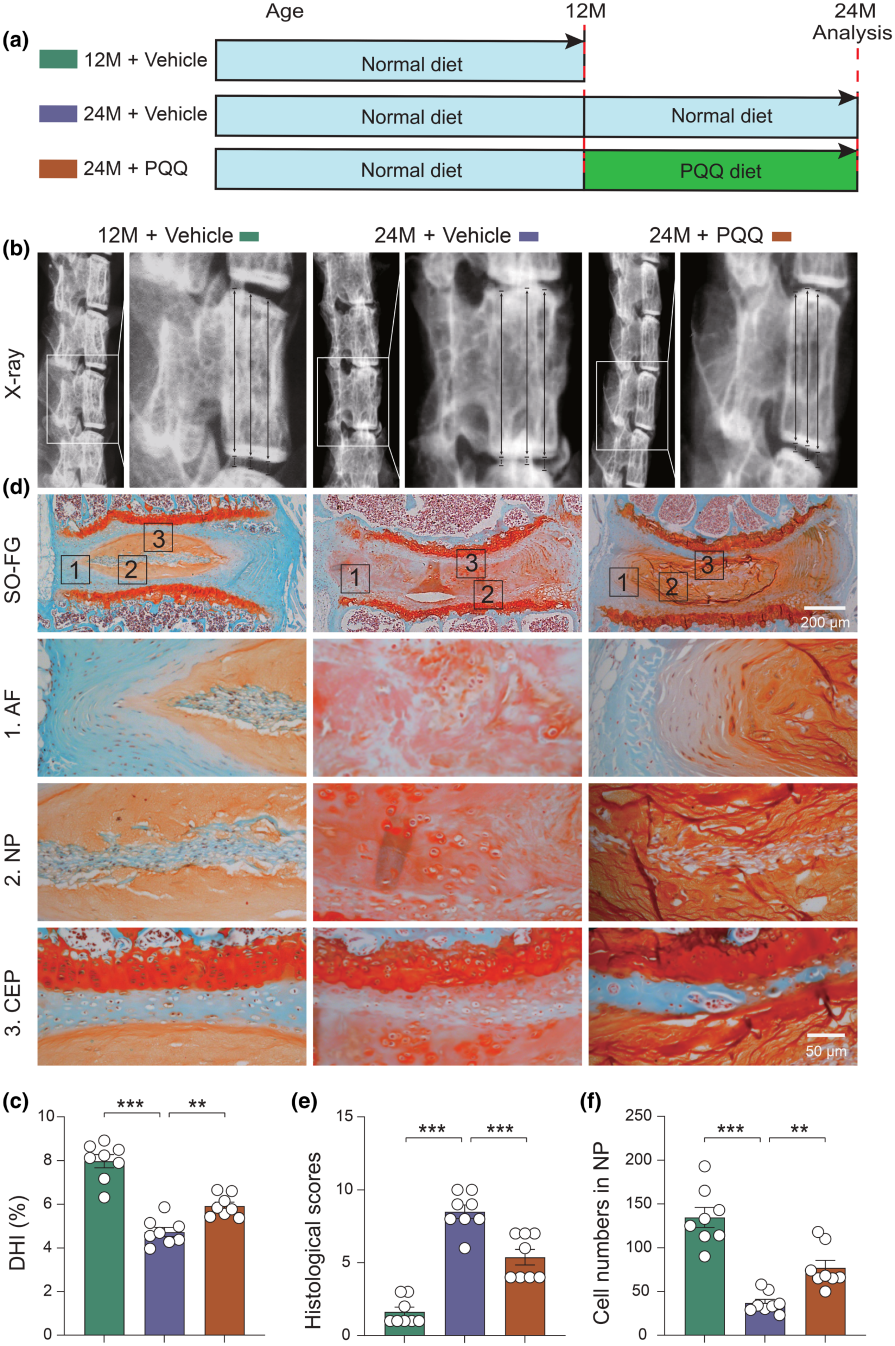
PQQ supplementation alleviates the IVDD phenotype in aging wild‐type mice. (a) Experimental design for investigating the effects of PQQ on natural aging‐induced IVDD; wild‐type mice at the age of 12 months were given the normal diet and PQQ diet (4 mg/kg standard feed) for 12 months, respectively. Control mice were given the normal diet. Mice were sacrificed and IVD phenotypes analyzed at indicated ages. (b) Representative X‐ray scans and (c) the changes in the disk height index (DHI). (d) Safranin O and Fast Green (SO&FG) staining. (e) Histological scores of lumbar IVDs in indicated groups of mice. (f) Cell number in NP tissues. One‐way ANOVA with Tukey's post hoc test. **p < 0.01, ***p < 0.001.

To investigate the potential of PQQ supplementation in preventing natural aging‐induced intervertebral disk degeneration (IVDD), 12‐month‐old wild‐type male mice were subjected to a PQQ‐containing diet (4 mg PQQ/kg diet) for 12 months, while the control groups (12‐ and 24‐month‐old male mice) received a standard diet (Figure 1a). Following sacrifice, IVDD phenotypes were evaluated using X‐ray and Safranin O‐fast green (SO&FG) staining. Compared to age‐matched littermates on a normal diet, PQQ supplementation significantly alleviated natural aging‐related IVDD in 24‐month‐old wild‐type mice, as evidenced by increased disk height index (DHI) percentage, reduced histological scores, and decreased cell numbers in the nucleus pulposus (NP) (Figure 1c–f). X‐ray scans in vivo revealed that PQQ supplementation effectively reversed age‐related kyphosis (Figure S1A). Notably, PQQ supplements, known to be safe in humans and approved by the U.S. Food and Drug Administration (FDA) (Akagawa et al., 2016; Office of Food Additive Safety (FHS‐200) Center for Food Safety and Applied Nutrition Food and Drug Administration, 2017), did not induce significant changes in total body weight, liver damage markers (e.g., aspartate aminotransferase [AST] and alanine aminotransferase [ALT]), and kidney damage markers (e.g., serum creatinine) in aged mice (Figure S1B–E). These findings suggest that PQQ supplementation effectively prevents natural aging‐related IVDD in wild‐type mice, presenting a promising option for mitigating natural aging‐induced IVDD.

### 3.2 Alleviation of cell senescence and ECM degradation by PQQ supplementation in IVD

**FIGURE 2:**
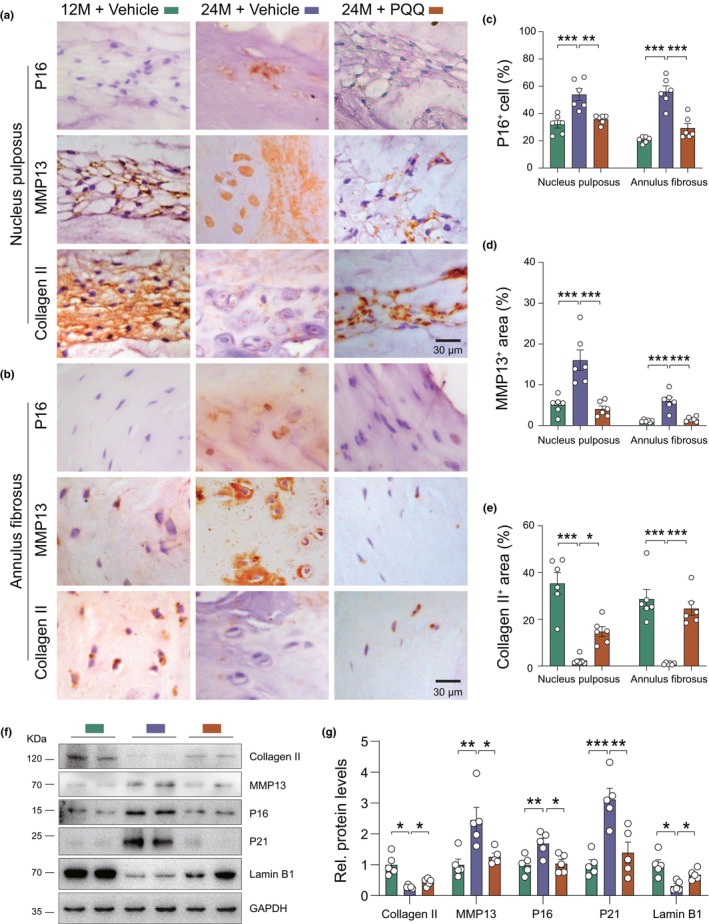
PQQ rescued aging‐induced ECM degradation and cellular senescence during aging. Representative images of IVD sections immunostained for P16, MMP13, and Collagen II in (a) NP and (b) AF. (c–e) Quantification of the percentage of (c) p16^+^ NPCs and AFCs, (d) MMP13^+^ area, and (e) Collagen II^+^ area in NP and AF. (f) Western blot detection and (g) the quantitative analysis of Collagen II, MMP13, P16, P21, and Lamin B1 protein levels in indicated groups. One‐way ANOVA with Tukey's post hoc test. *p < 0.05, **p < 0.01, ***p < 0.001.

To elucidate the mechanisms contributing to the alleviation of aging‐related IVDD in PQQ‐supplemented aged mice, we examined oxidative stress, cellular senescence, and extracellular matrix (ECM) homeostasis. Immunohistochemistry revealed disruptions in ECM homeostasis in the lumbar nucleus pulposus and anulus fibrosus of 24‐month‐old mice, characterized by decreased collagen type II (Collagen II) and increased matrix metalloproteinase 13 (Mmp13) expression. Pyrroloquinoline quinone treatment significantly rescued these alterations (Figure 2a,b,d–g). Elevated oxidative damage marker (e.g., 8‐OHdG), cellular senescence markers (e.g., P16, P21 and Lamin B1), and senescence‐associated secretory phenotype (SASP)‐related markers (IL‐1β and IL‐6) observed in 24‐month‐old mice were also ameliorated by PQQ supplementation (Figure 2a–c,f–g, Figure S2A–G). These results demonstrate that PQQ supplementation prevents natural aging‐related IVDD by inhibiting oxidative stress, cellular senescence, and ECM degradation. Notably, the TUNEL assay revealed a significant decrease in apoptotic cells in the NP tissues of aged mice treated with PQQ as compared to the control group (Figure S3A,B), indicating that PQQ also partly reduces cell apoptosis in NP tissues with aging via inhibiting oxidative stress.

### 3.3 Inhibition of ROS accumulation and cell senescence by PQQ in IL‐1β‐treated NPCs

**FIGURE 3:**
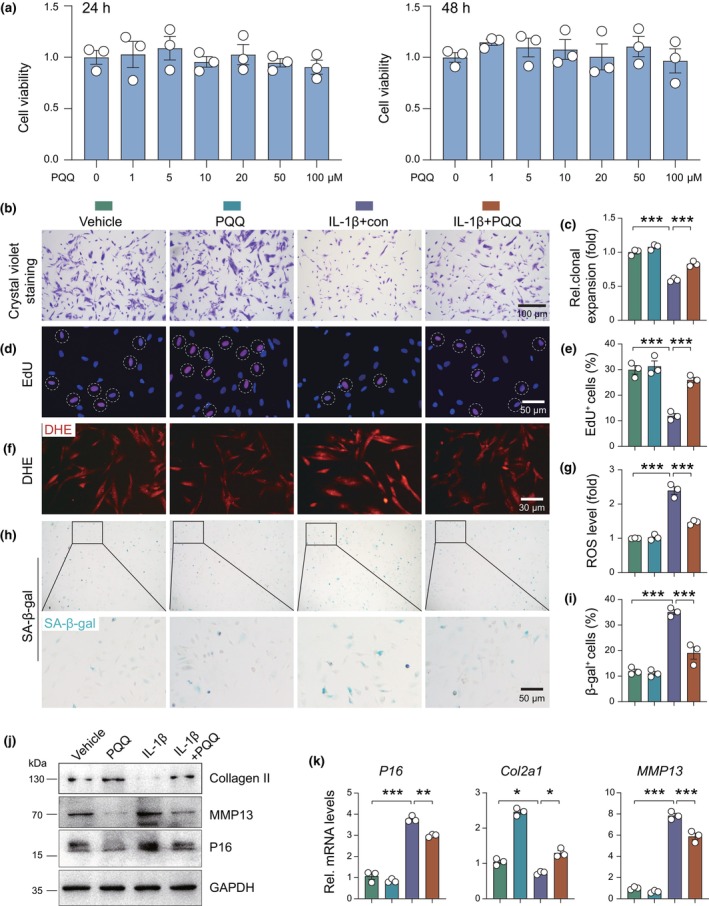
Effects of PQQ on ROS accumulation, cell proliferation, and senescence phenotype in IL‐1β‐treated human NPCs. (a) Human NPCs viability was determined using CCK8 assay following PQQ (0, 1, 5, 10, 20, 50, 100 μM) treatment for 24 and 48 h. (b) Crystal violet staining and (c) relative clonal expansion analysis. (d) EdU staining and (e) the quantitative analysis of EdU^+^ NPCs. (f) ROS levels determined using dihydroethidium (DHE) staining in vitro and (g) the quantitative analysis of ROS levels. (h) SA‐β‐gal staining and (i) the quantitative analysis of SA‐β‐gal^+^ NPCs. (j) Western blot detection and (k) qPCR detection of P16, Col2a1, and MMP13 in indicated groups. One‐way ANOVA with Tukey's post hoc test. *p < 0.05, **p < 0.01, ***p < 0.001.

To assess the impact of PQQ on interleukin‐1β (IL‐1β)‐induced ECM degradation and cellular senescence in human nucleus pulposus cells (NPCs) in vitro, we treated NPCs with PQQ for 24 or 48 h. Pyrroloquinoline quinone exhibited no significant cytotoxicity at concentrations ranging from 1 to 100 μM (Figure 3a). Crystal violet staining and EdU staining results showed that the proliferation of NPCs was decreased by IL‐1β treatment, while PQQ significantly increased the ratio of proliferating cells in IL‐1β‐treated NPCs as compared with vehicle‐treated control (Figure 3b–e). In addition, DHE staining showed that ROS levels in NPCs were significantly decreased in PQQ‐treated NPCs relative to vehicle‐treated controls in the presence of IL‐1β (Figure 3f,g). SA‐β‐gal staining showed that SA‐β‐gal^+^ senescent cells were significantly lower in PQQ‐treated NPCs than in vehicle‐treated controls in the presence of IL‐1β (Figure 3h,i). Additionally, PQQ treatment reversed IL‐1β‐induced alterations in senescence markers (p16), ECM proteins (MMP13, Collagen II), and cell proliferation (EdU staining) in NPCs (Figure 3j,k). Furthermore, to assess the impact of PQQ on NPCs under short‐term oxidative stress induction, human NPCs were exposed to IL‐1β for specified durations in the presence or absence of PQQ. Results revealed that the mRNA expression levels of Nrf2 target genes, including HO‐1, Gclc, and Gclm, were significantly increased following 3 and 6 h of IL‐1β treatment (Figure S4A). However, the mRNA and total protein levels of Nrf2 remained unchanged (Figure S4B). Notably, the nuclear level of Nrf2 was significantly increased upon brief IL‐1β exposure (Figure S4B). Furthermore, the short‐term treatment of IL‐1β had no significant impact on the viability of human NPCs, and concurrent treatment with PQQ had no effect on cell viability during the 24‐h IL‐1β treatment period (Figure S4C). These findings suggest that PQQ protects against IL‐1β‐induced ECM degradation in NPCs by attenuating ROS accumulation and cellular senescence.

### 3.4 Binding and disruption of Keap1–Nrf2 complex by PQQ

**FIGURE 4:**
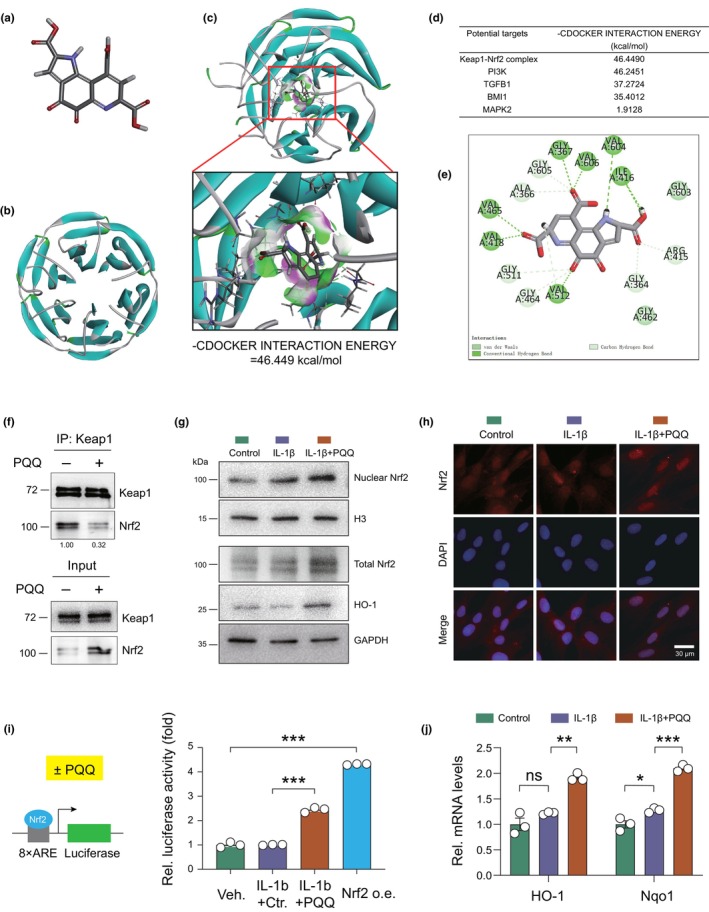
PQQ binds to and disrupts Keap1–Nrf2 complex resulting in Nrf2–ARE activation. (a) The model of PQQ. (b) The ribbon model of the Keap1–Nrf2 complex. (c) The interaction between PQQ and Keap1–Nrf2 complex; ‐CDOCKER energy is 46.4490 kcal/mol. (d) Other potential targets of PQQ. (e) 2D binding model between PQQ and Keap1–Nrf2 complex, and the molecules of Keap1–Nrf2 complex that interact with PQQ through van der Waals, salt bridges, alkyl and hydrogen bonds. (f) The interaction between Keap1 and Nrf2 in PQQ‐treated human NPCs relative to vehicle‐treated control was determined using endogenous Co‐IP. (g) Western blot detection of nuclear‐Nrf2, total‐Nrf2, and HO1 in human NPCs following PQQ treatment for 24 h in the presence and absence of IL‐1β. (h) IF detection of Nrf2 in indicated groups of human NPCs. (i) Relative luciferase activity driven by ARE in human NPCs following PQQ treatment for 24 h in the presence and absence of IL‐1β were determined using dual luciferase assay. (j) qPCR detection of HO‐1 and Nqo1 in indicated groups. One‐way ANOVA with Tukey's post‐hoc test. *p < 0.05, **p < 0.001, ***p < 0.001. ns: not significant.

To further explore the mechanism underlying the effect of PQQ on oxidative stress and cellular senescence, we applied molecular docking analysis to screen candidate proteins that might be regulated by PQQ. Before the molecular docking analysis, the structure of PQQ was established in Discovery Studio 2016; potential targets of PQQ, such as Keap1–Nrf2 complex, PI3K, TGFB1, BMI1, and MAKP2, were downloaded from the Protein Data Bank (PDB) (Figure 4a,b). Subsequently, molecular docking analysis was adopted to evaluate the affinity of the PQQ against potential targets. Results showed that PQQ against the Keap1–Nrf2 complex acquired the relatively highest ‐CDOCKER energy score (46.4490 kcal/mol), which means that PQQ has the highest affinity with Keap1–Nrf2 complex (Figure 4c,d). Nrf2 is an important antioxidant transcription factor, and Nrf2 forms hydrogen bonds with Keap1 residues to maintain its stability. Upon exposure to oxidative stress, Nrf2 might dissociate from Keap1, enter the nucleus and bind to antioxidant‐responsive elements, thus upregulating functional genes, such as HO1. Previous studies have reported that Nrf2 activation can inhibit cellular senescence (Shao et al., 2021). To confirm the ability of PQQ to activate Nrf2, a 2D binding model was adopted to exhibit the binding sites of PQQ and Keap1–Nrf2 complex. Interaction with specific residues (VAL512, VAL418, VAL465, VAL606, VAL604, ILE416, GLY367) suggests the potential of PQQ to disrupt the Keap1–Nrf2 complex, promoting Nrf2 translocation to the nucleus (Figure 4e). Co‐immunoprecipitation assays demonstrated reduced binding of Nrf2 to Keap1 in PQQ‐treated NPCs as compared to vehicle‐treated control (Figure 4f). To further confirm whether the Nrf2 pathway is involved in the effects of PQQ on senescence in IL‐1β‐treated NPCs, we firstly separated nucleus from the NPCs and evaluated the level of Nrf2 in the nucleus and the level of its downstream effector protein HO1 in whole NPCs in the presence of PQQ using Western blot. Results showed that PQQ increased the expression of Nrf2 in the nucleus and the expression of Nrf2 and HO1 in the whole cells (Figure 4g). Moreover, immunofluorescence results confirmed that PQQ increased the nucleus accumulation of Nrf2 (Figure 4h). Furthermore, ARE‐driven luciferase activity was also significantly increased in PQQ‐treated NPCs in indicated times (Figure 4i), and qPCR assay further confirmed that Nrf2 downstream targets HO‐1 and Nqo1 were significantly increased at the mRNA level in PQQ‐treated NPCs as compared with control (Figure 4j). These results indicate that PQQ disrupts the Keap1‐Nrf2 complex, activating Nrf2‐ARE signaling.

### 3.5 Nrf2 activation inhibits ECM degradation by upregulating Wnt5a in NPCs

**FIGURE 5:**
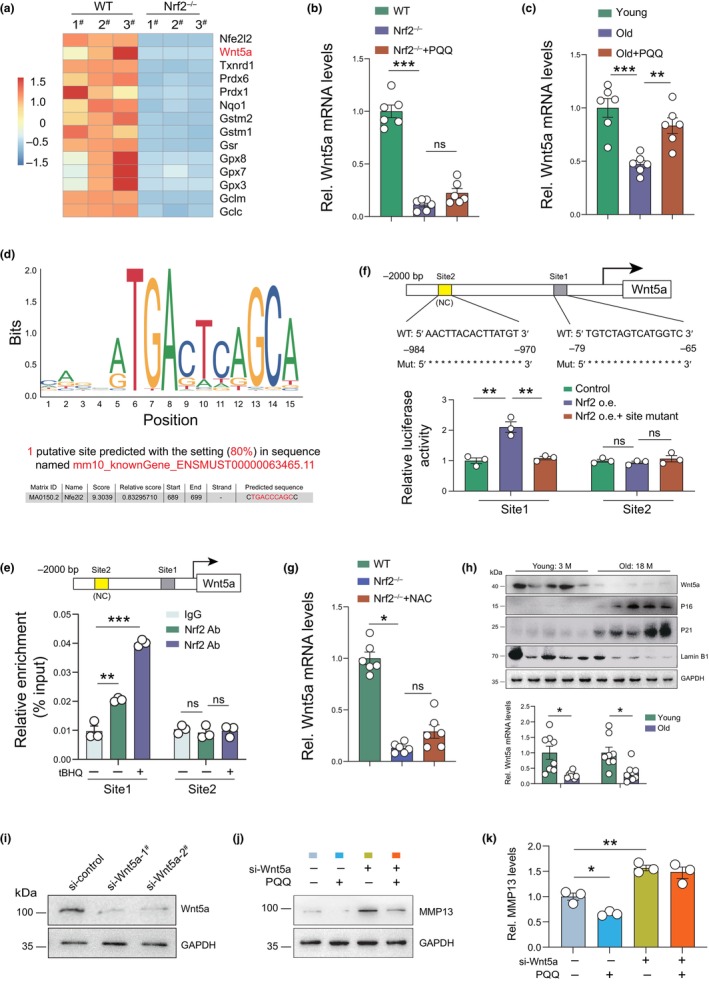
Nrf2 activation inhibits ECM degradation by upregulating Wnt5a in NPCs. (a) RNA‐seq heatmap showing the mentioned genes expressed in BM‐MSCs isolated from WT and Nrf2^−/−^ mice. (b) The mRNA levels of Wnt5a in NPCs from 18‐month‐old WT, Nrf2^−/−^ mice and PQQ‐treated Nrf2^−/−^ mice. (c) The mRNA levels of Wnt5a in mouse NPCs from young (3‐month‐old), old (18‐month‐old), and PQQ‐treated 18‐month‐old WT mice. (d) A predictive Nrf2‐binding element in mouse Wnt5a promoter region. (e) Chromatin immunoprecipitation (ChIP) with Nrf2 antibody or IgG antibody were performed in vehicle‐ or tBHQ‐treated mouse NPCs and relative enrichment of Wnt5a promoter was determined using qPCR assay. (f) Mouse Wnt5a promoter or Wnt5a promoter mutant Luc‐plasmids were transfected into mouse NPCs following lentivirus‐mediated Nrf2 overexpression and relative luciferase activity were analyzed after 48 h. (g) The mRNA levels of Wnt5a in mouse NPCs from 3‐month‐old WT, Nrf2^−/−^ mice, and NAC‐treated Nrf2^−/−^ mice. (h) The protein and mRNA levels of Wnt5a in NP tissues from young (3‐month‐old) and old (18‐month‐old) wild‐type mice. Western blot detection of P16, P21, and Lamin B1 was used as cell senescence markers. (i) Knock‐down efficiency of si‐Wnt5a determined using Western blot. (j) Western blot detection of MMP13 in vehicle‐ or PQQ‐treated human NPCs in the presence or absence of si‐Wnt5a. (k) Quantitative analysis of MMP13 in (j). One‐way ANOVA with Tukey's post hoc test. *p < 0.05, **p < 0.01, ***p < 0.001. ns: not significant.

We next examined whether PQQ‐mediated Nrf2 activation can directly inhibit matrix degradation independent of its role in stress response regulation. Mesenchymal stem cells (MSCs) have been reported to have the potential to differentiate into IVD‐like cells, and recent work indicate that MSCs transplantation might be a suitable option for the treatment of IVDD (Ohnishi et al., 2023). Therefore, we analyzed RNA‐seq data from primary bone marrow‐derived mesenchymal stem cells (BM‐MSCs) and found a significant decrease in wingless‐type MMTV integration site family, member 5A (Wnt5a) expression in Nrf2^−/−^ cells (Figure 5a). The decreased expression of Wnt5a was also detected in human NPCs with siRNA‐mediated Nrf2 knockdown (Figure S5A). Wnt5a has been reported to suppress the activation of NF‐κB signaling and its downstream genes including MMP13, thus inhibiting ECM degradation and IVDD (Li et al., 2018). Real‐time qPCR results PQQ treatment increased Wnt5a expression in aged wild‐type NPCs but not in age‐matched Nrf2^−/−^ NPCs, indicating Nrf2 dependency (Figure 5b,c). Based on these results, we speculated that PQQ might regulate the transcription of Wnt5a through Nrf2. A predicted ARE was detected in the promoter of Wnt5a (Figure 5d), containing the sequence TGACCCAGC, coinciding with the consensus ARE motif (TGACNNNGC). Chromatin immunoprecipitation‐quantitative PCR (ChIP‐qPCR) assay of NPCs confirmed that Nrf2 bound to this putative region of Wnt5a under physiological conditions and this binding was significantly strengthened when the cells were treated with tert‐butylhydroquinone (tBHQ, a classic Nrf2 activator) (Figure 5e). Dual luciferase assays showed that luciferase activity driven by a Wnt5a promoter containing the predicted ARE was significantly increased following Nrf2 overexpression, while this effect was abolished when the predicted ARE was mutated (Figure 5f). Furthermore, the antioxidant reagent NAC did not rescue the decreased Wnt5a mRNA level in Nrf2^−/−^ NPCs (Figure 5g), indicating that the increased Wnt5a induced by Nrf2 activation was independent of decreased oxidative stress. Meanwhile, we detected Wnt5a expression in mouse IVD tissues and found that the expression level of Wnt5a was also significantly lower in aging IVD tissues than in young controls (Figure 5h). Moreover, Wnt5a knockdown significantly increased MMP13 production in mouse NPCs in vitro. Moreover, Wnt5a knockdown reversed the inhibitory effects of Nrf2 activation (PQQ) on MMP13 production in NPCs (Figure 5i–k). These findings indicate that Nrf2 activation inhibits MMP13 production by transcriptionally upregulating Wnt5a.

### 3.6 Nrf2 is essential for PQQ‐mediated inhibition of aging‐related IVDD

**FIGURE 6:**
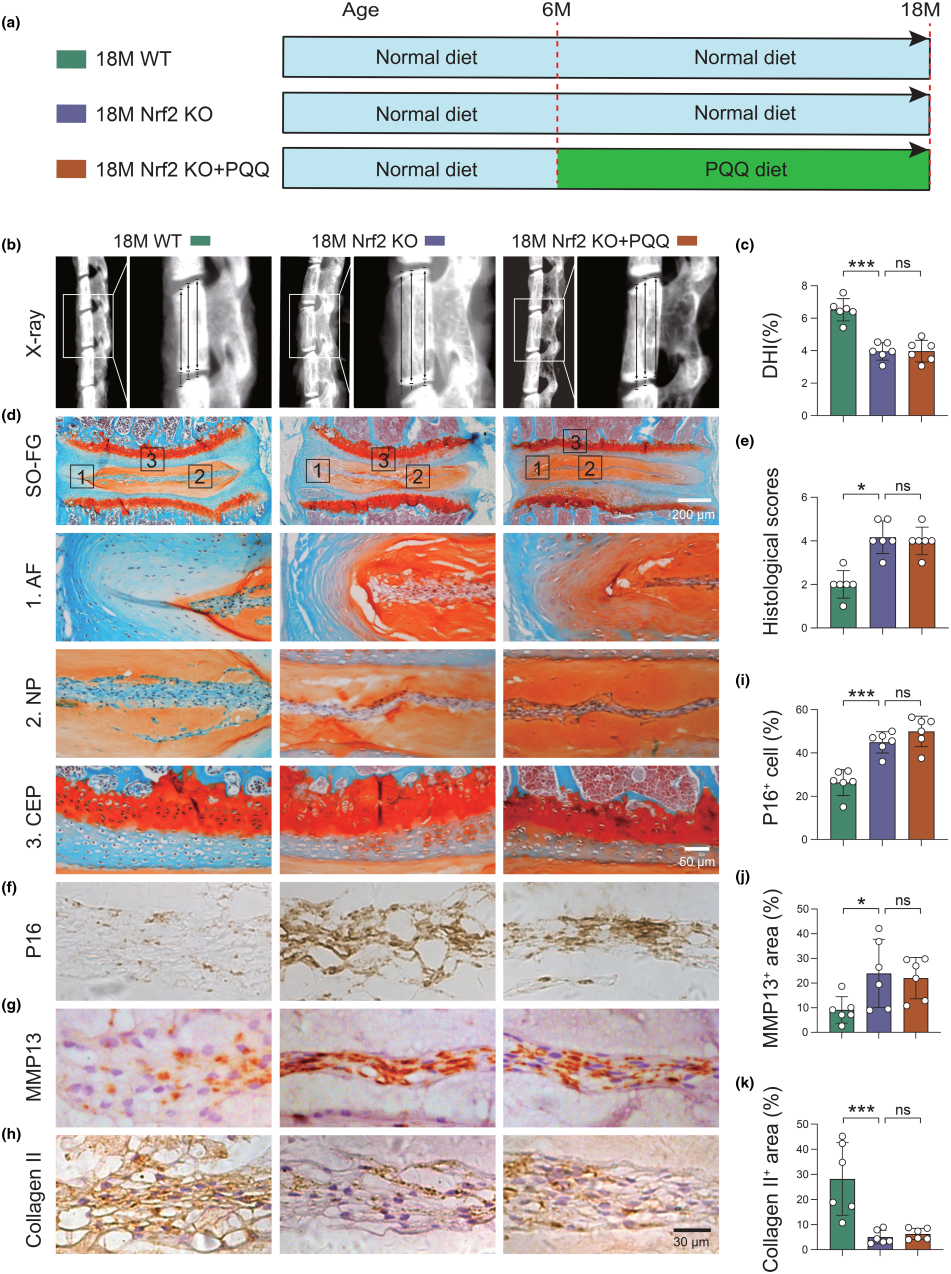
Nrf2 knockout blunts the preventing effects of PQQ on aging‐related IVDD. (a) Experimental design for investigating the effects of PQQ on Nrf2 deficiency‐induced IVDD; Nrf2‐deficient mice at the age of 6 months were given the normal diet and PQQ‐containing diet (4 mg/kg standard feed) for 12 months, respectively. Six‐month‐old control mice were given the normal diet. (b) Representative X‐ray scans and (c) the quantitative analysis of DHI. (d) Safranin O and Fast Green (SO&FG) staining and (e) histological scores of lumbar IVD in indicated groups of mice. (f–h) Representative images of lumbar IVD sections immunostained for (f) P16, (g) MMP13, and (h) Collagen II in NP tissues. (i–k) Quantification of the percentage of (i) p16^+^ NPCs, (j) MMP13^+^ area, and (k) Collagen II^+^ area in NP tissues. One‐way ANOVA with Tukey's post hoc test. *p < 0.05, ***p < 0.001. ns: not significant.

In order to determine whether Nrf2 is the key mediator of the inhibitory effects of PQQ on cellular senescence and aging‐related IVDD, 6‐month‐old wild‐type and Nrf2^−/−^ male mice were fed PQQ‐ or vehicle‐containing diets for 12 months, and intervertebral disk phenotypes were analyzed using μCT and histomorphometry at the age of 18 months (Figure 6a). We firstly found that aged Nrf2^−/−^ mice exhibited more severe aging‐related IVDD phenotypes than age‐matched WT mice, including decreased DHI and increased histological score, DNA damage, NPCs senescence, SASP, and ECM degradation (Figure 6b–k, Figure S6A–E). Furthermore, DHI, histological score, cellular senescence, SASP, and ECM degradation were significantly rescued in PQQ‐supplemented wild‐type mice as described in Figures 1 and 2; however, these alterations were not significantly changed in PQQ‐supplemented Nrf2^−/−^ mice as compared with vehicle‐treated Nrf2^−/−^ mice (Figure 6b–k, Figure S6A–E). Similarly, the inhibitory effects of PQQ on cell proliferation, ROS accumulation, and cellular senescence were also significantly blocked in Nrf2‐knockdown NPCs as compared to control group (Figure S7A–J). These results emphasize the indispensable role of Nrf2 in mediating the protective effects of PQQ against aging‐related IVDD, both in vivo and in vitro.

## 4 DISCUSSION

Pyrroloquinoline quinine (PQQ), known for its potent antioxidant capacity and availability in various foods, has received approval from the Food and Drug Administration (FDA) as a dietary supplement. This makes PQQ an attractive candidate for mitigating aging‐related diseases, including intervertebral disk degeneration (IVDD). Previous studies have demonstrated the efficacy of PQQ in preventing diverse conditions such as osteoporosis, renal fibrosis, and reproductive dysfunction (Dai et al., 2022; Geng et al., 2019; Qu et al., 2022; Wang, Zhang, et al., 2023; Wu et al., 2017). Despite these findings, its potential in alleviating natural aging‐related IVDD and the specific underlying mechanisms remain elusive. Here, our study sheds light on the preventive effects of dietary PQQ supplementation on oxidative stress, cellular senescence, and extracellular matrix (ECM) degradation in nucleus pulposus cells (NPCs) through Keap1–Nrf2 signaling‐mediated stress response and Wnt5a upregulation, thus preventing aging‐related IVDD (Graphical abstract).

PQQ was identified as a redox cofactor with strong radical‐scavenging activity, and it has been reported effective in alleviating several disorders (Jonscher et al., 2017; Stites et al., 2006). For instance, our previous studies have demonstrated that PQQ can prevent oophorectomy‐ or aging‐induced osteoporosis (Geng et al., 2019; Li et al., 2023). In this study, we intend to investigate the effects of PQQ on aging‐related IVDD. Here, we administered PQQ to wild‐type mice from 12 months of age for 12 months, and found that PQQ alleviated natural aging‐induced IVDD. Our recent findings indicate that PQQ could be a potential drug beneficial for the prevention and treatment of aging‐related osteoporosis (Li et al., 2023). Moreover, we demonstrated that long‐term PQQ supplementation did not lead to body weight loss or other organ‐damaging side effects, which is consistent with a previous work with short‐term PQQ supplementation (Harris et al., 2013). This suggests that PQQ appears to be an attractive option for delaying the progression of aging‐related IVDD in humans, and this needs to be investigated in future studies.

Aging‐induced ROS accumulation and cellular senescence have been recognized as the key driving forces in the pathogenesis of several aging‐related diseases, including IVDD. Indeed, previous studies identified the increase of senescent NPCs in IVD with aging; meanwhile, depleting P16^+^ cells or eliminating senescent cells using senolytics have been reported to effectively alleviate unloading‐ or aging‐related IVDD (Novais et al., 2021; Patil et al., 2019). In this study, long‐term PQQ supplementation alleviated aging‐related IVDD by reducing oxidative damage, cellular senescence, and SASP in vivo. Besides, PQQ treatment decreased oxidative damage, inhibited cellular senescence, and increased cell proliferation and ECM production of NPCs in vitro in the presence of IL‐1β. Nrf2 is the key master of redox, and we have recently reported that PQQ exerts antioxidant function by activating Nrf2 signaling (Li et al., 2023). Therefore, the protective effects of PQQ on IVDD appears to be consistent with the crucial role for Nrf2‐antioxidant signaling for redox homeostasis and cell survival (Yoon et al., 2016). Indeed, we here found that PQQ‐mediated stress response, decreased oxidative stress, and cellular senescence in NPCs in vitro were largely blocked following Nrf2 knockdown. These findings indicate that Nrf2 is essential for the inhibitory effects of PQQ on oxidative stress and cellular senescence. Previous studies (Sánchez‐de‐Diego et al., 2021) and the present study indicate that Nrf2 exerts protective effects in response to aging‐induced stress in IVD; however, Nrf2 global change might indirectly regulate IVD homeostasis, and thus targeted activation or deficiency of Nrf2 in IVD‐related cells in aged mice is needed to test this hypothesis.

Here, we also explored the mechanism whereby PQQ disrupted the Keap1–Nrf2 complex, activating Nrf2–ARE signaling. The modification of cysteine residues and conformational change in Keap1 can be induced by oxidative stress, reducing Nrf2 ubiquitination and degradation. Additionally, mounting evidence indicates that there is cross‐talk between Keap1–Nrf2 and other proteins, such as aPKCι, iASPP2, p62, and Nestin (Ge et al., 2017; Komatsu et al., 2010; Tian et al., 2019; Wang, Lu, et al., 2019), which can disrupt the normal Keap1–Nrf2 interaction and facilitate Nrf2‐mediated cellular redox homeostasis. Here, we found that PQQ preferably binds to Keap1–Nrf2 complex using a small molecule drug binding protein prediction site (http://www.lilab‐ecust.cn/pharmmapper/). We further used Autodocking software to verify the direct interaction between PQQ and Keap1–Nrf2 complex. In addition, we here demonstrated that PQQ treatment can disrupt the interaction between Nrf2 and Keap1, causing the translocation of Nrf2 into the nucleus and increasing the transcriptional activity of Nrf2. Overall, these data provide the mechanism by which PQQ can activate Nrf2–ARE signaling through disrupting Keap1–Nrf2 complex and releasing Nrf2.

Previous studies, as well as our present study, have demonstrated that PQQ possesses the ability to neutralize two prominent causes of mitochondrial dysfunction: superoxide and hydroxyl radicals. It is worth noting that while mitochondria are a common source of ROS, our investigation suggests that PQQ may also contribute to the reduction of ROS through its effects on mitochondrial function and biogenesis. Specifically, PQQ has been found to protect HK‐2 cells against high glucose‐induced mitochondrial ROS through the Sirt3 and PI3K/Akt/FoxO3a signaling pathway (Wang, Li, et al., 2019). Additional studies have revealed that PQQ enhances mitochondrial biogenesis by stimulating the phosphorylation of cAMP response element‐binding protein (CREB), as well as the activation of Sirt1/Sirt3, thereby promoting the expression of peroxisome proliferator‐activated receptor‐γ coactivator‐1α (PGC‐1α) and nuclear respiratory factors (NRF) (Chowanadisai et al., 2010; Zhang et al., 2015). Furthermore, PQQ acts as a novel activator of the nuclear factor erythroid 2‐related factor‐2 (Nrf2), which in turn maintains mitochondrial homeostasis by activating downstream genes associated with various aspects of mitochondrial function, including biogenesis, integrity, and mitophagy (Dinkova‐Kostova & Abramov, 2015; Zhang et al., 2024). Thus, it can be inferred that the activation of Nrf2‐induced mitochondrial homeostasis may also contribute to PQQ's ability to counteract oxidative stress.

Although previous studies have reported that Nrf2 activation can prevent IVDD by inhibiting oxidative stress and extracellular matrix degradation, whether Nrf2 can directly inhibit ECM degradation independent of oxidative stress‐induced MMP production in NPCs is unclear. Here, we performed RNA‐seq assay and found that Wnt5a was significantly decreased in Nrf2^−/−^ NPCs and MSCs apart from other known Nrf2 downstream targets. Wnt5a was elevated in degenerated human NP tissue and in TNFα‐treated NPCs (Li et al., 2018), and it has been reported to prevent puncture‐induced IVDD in vivo through suppressing NF‐κB signaling and subsequent MMPs expression (Li et al., 2018). Consistently, we found that Wnt5a knockdown in NPCs increased MMP13 production; of note, Wnt5a knockdown significantly blocked the inhibitory effects of Nrf2 activation on MMP13 expression in NPCs. Furthermore, we used a website (https://jaspar.genereg.net/) to predict possible binding sites of Nrf2 in the Wnt5a promoter and found one classic ARE site in the Wnt5a promoter. We found that Nrf2 can transcriptionally upregulate Wnt5 by binding to its promoter, and this effect of Nrf2 on Wnt5a appears to be independent of oxidative stress regulation as the antioxidant NAC does not rescue the downregulation of Wnt5 caused by Nrf2 deficiency. In conclusion, we report here, for the first time, that Nrf2 can bind to the promoter region of Wnt5a to regulate Wnt5a expression, antagonize MMP13, and inhibit ECM degradation, providing a novel mechanism for Nrf2 activation in directly inhibiting ECM degradation. Considering that MSCs and its exosomes have great potential in the treatment of IVDD, and the Keap1–Nrf2–Wnt5a axis may also function in MSCs, we speculate that MSCs and its exosomes with targeted activation of Nrf2 may be attractive options for the treatment of aging‐related IVDD. However, this needs to be further investigated in future studies.

Here, we report for the first time that aged Nrf2 deficiency accelerated the progression of aging‐related IVDD phenotypes. Consistently, Nrf2 was progressively decreased in human NP tissue samples of patients with increasing degrees of IVD degeneration (Tang et al., 2019). Moreover, Nrf2‐signaling activation has recently been shown to be effective in alleviating IVDD phenotypes in puncture‐induced IVDD models (Huang et al., 2023; Ma et al., 2022). To further determine whether Nrf2 could serve as a key target of PQQ in correcting natural aging‐induced IVDD, 6‐month‐old WT and Nrf2^−/−^ mice were supplemented with PQQ in vivo and their phenotypes were analyzed. In this study, PQQ supplementation corrected aging‐related IVDD in aging wild‐type mice; however, these responses were largely blocked in Nrf2^−/−^ mice. In addition, we found that PQQ decreased oxidative stress, cellular senescence, SASP, and ECM degradation in IVD; however, these responses were significantly blocked by Nrf2 deficiency. Overall, these results suggest that Nrf2 is essential for the role of PQQ in alleviating aging‐induced IVDD by increasing the oxidative stress response and reducing MMP13 production in NPCs.

One limitation of our study is the focus on the role of PQQ in regulating oxidative stress and identifying the Keap1–Nrf2–Wnt5a pathway in aging‐related IVDD. Future investigations should explore the possibility of PQQ affecting other organs through specific targets and the development of drug delivery systems to ensure precise targeting of the intervertebral disk. Additionally, the study did not examine whether PQQ supplementation can reverse puncture‐induced IVDD, and future work should evaluate the safety and efficacy of PQQ in age‐related IVDD in humans.

In conclusion, our study unravels the potential of PQQ in disrupting the Keap1–Nrf2 complex, activating Nrf2‐ARE signaling, and transcriptionally upregulating Wnt5a. This intricate interplay results in enhanced cellular stress response, reduced cellular senescence, and inhibition of ECM degradation, ultimately preventing aging‐related IVDD. These findings position PQQ as a promising option for the treatment and prevention of aging‐related IVDD.

## AUTHOR CONTRIBUTIONS

R.Y. and D.M. conceived the project. Q.X. and J.L. performed most of the experiments, analyzed, and compiled the data. R.Q., M.L., Y.L., J.Z., and R.W. helped with experiments. R.Y., D.M., and D.G. participated in writing or editing the paper.

## FUNDING INFORMATION

This work was supported by grants from the National Natural Science Foundation of China (81730066 to DM) and the Canadian Institutes of Health Research (PJT‐152963 to DG).

## CONFLICT OF INTEREST STATEMENT

None declared.

## Supporting information


Figures S1–S7

Tables S1–S3

## Data Availability

All data and materials used in the study are available to any researcher for purposes of reproducing or extending the analysis.
