# Supplementary figures and images for: Nrf2 activation by pyrroloquinoline quinone inhibits natural aging‐related intervertebral disk degeneration in mice
Source: Aging Cell. 2024 May 23;23(8):e14202. doi: 10.1111/acel.14202 (PMC11320358; doi:10.1111/acel.14202)

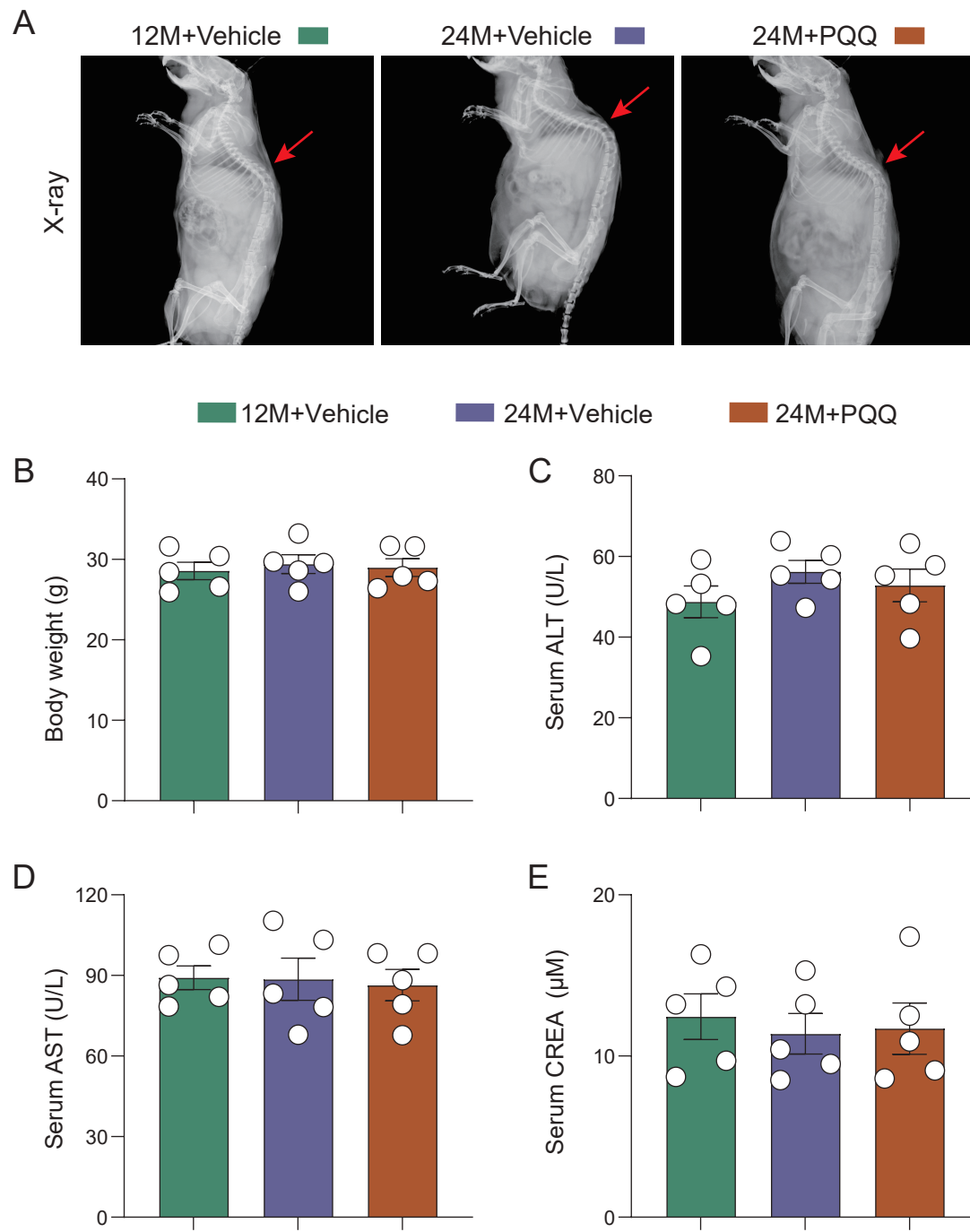

Fig. S1

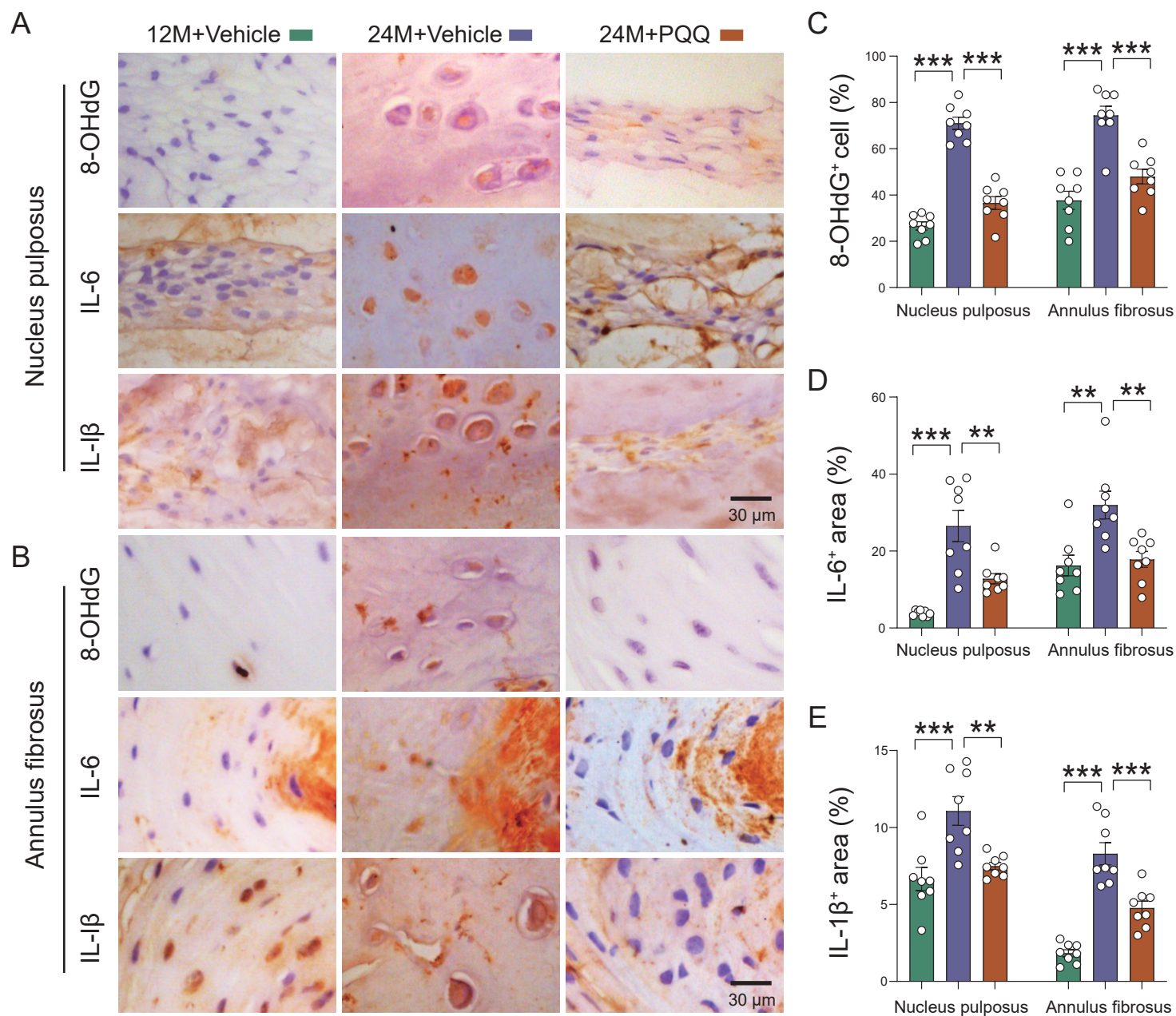

Fig. S2

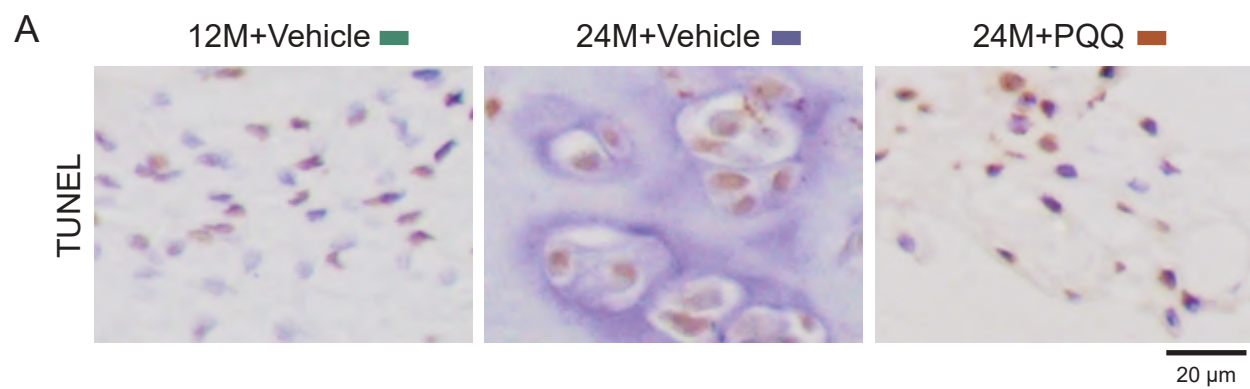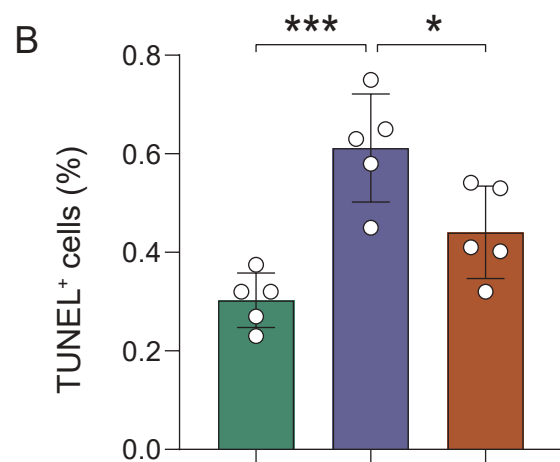

Fig.S3

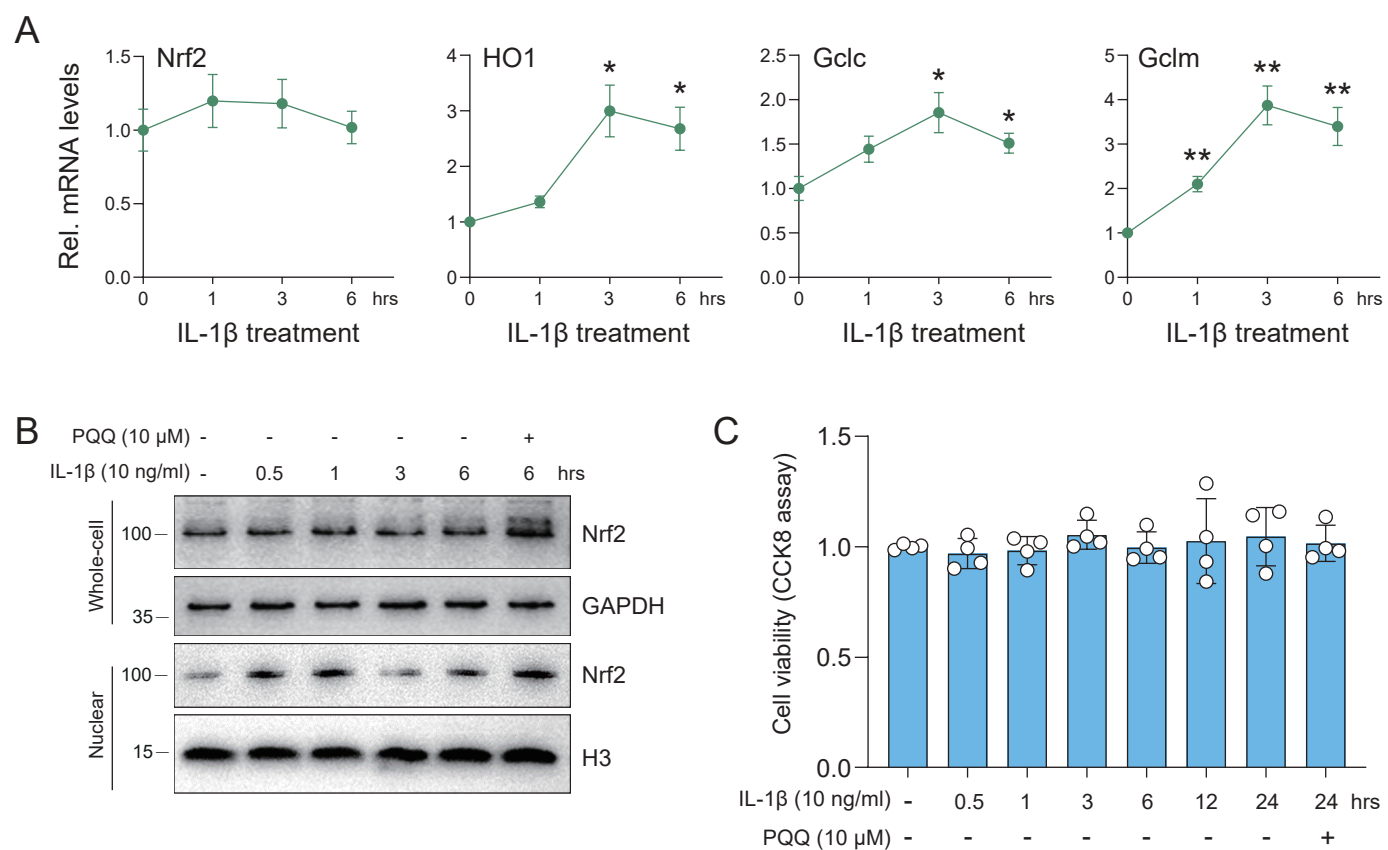

Fig. S4

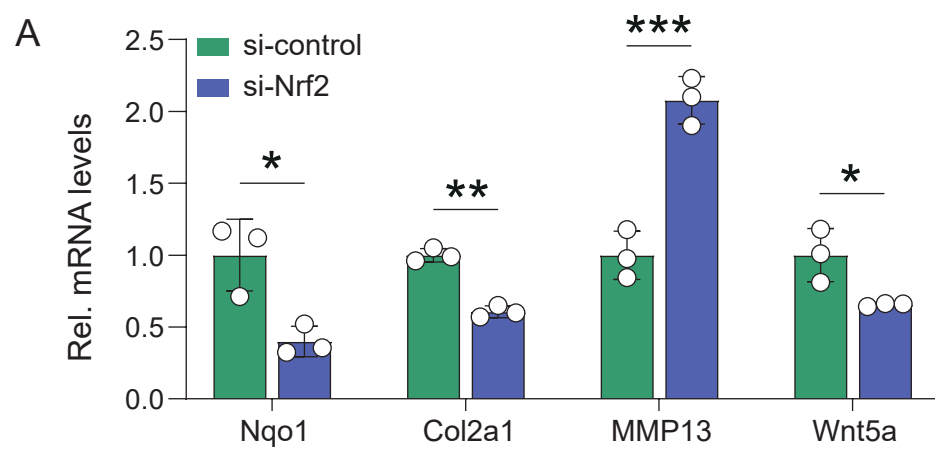

Fig. S5

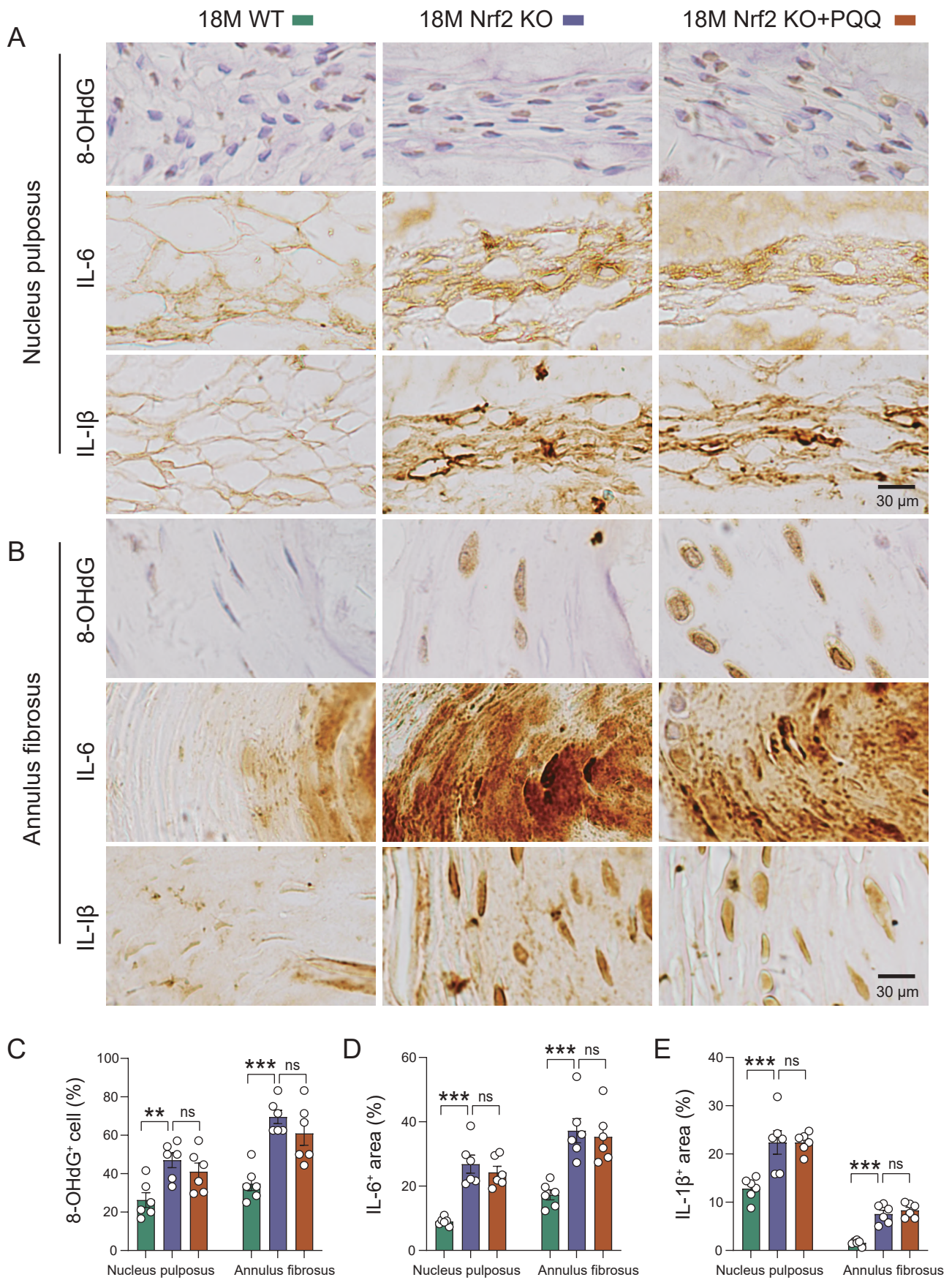

Fig. S6

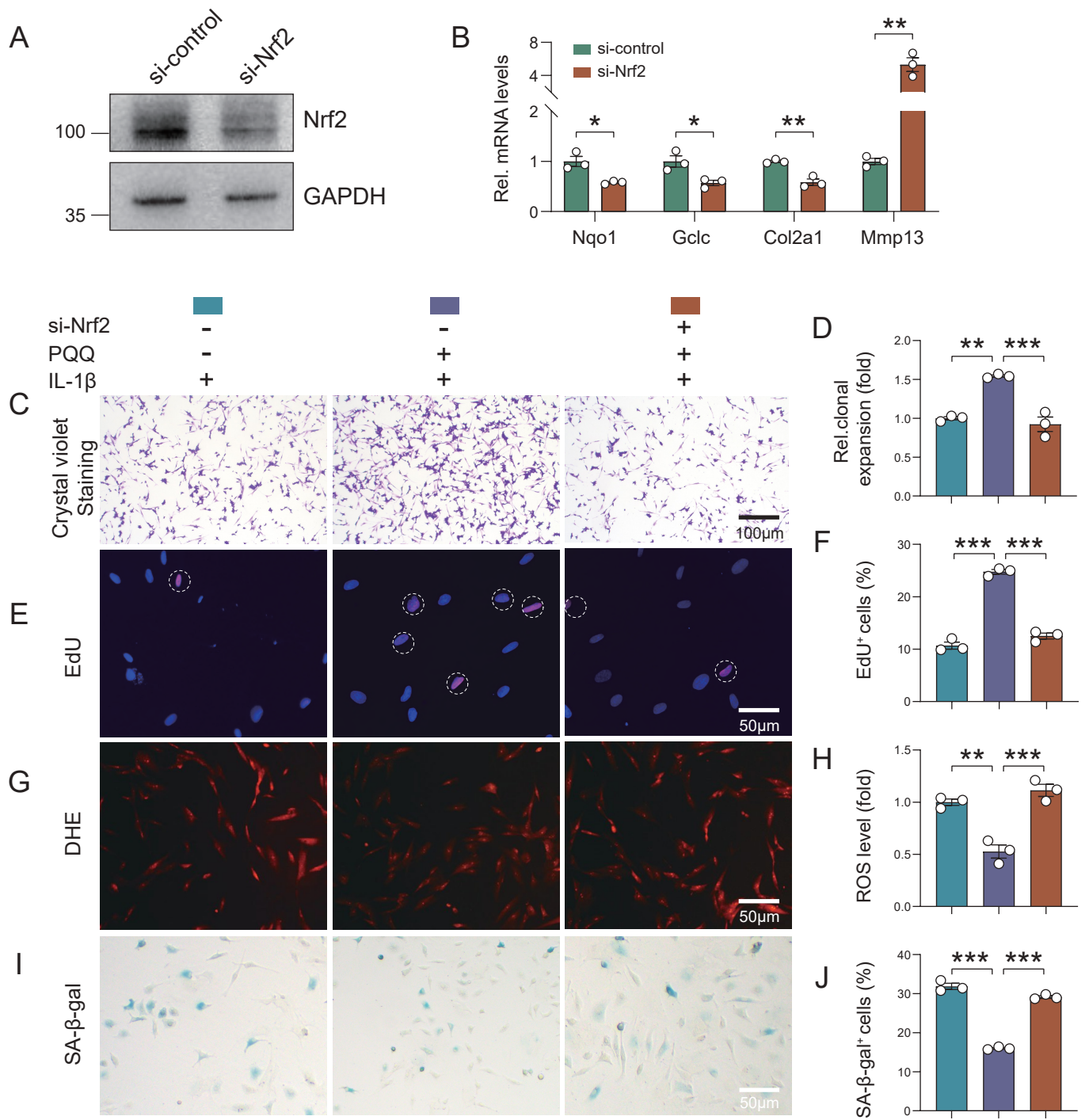

Fig.S7

Supplement: Supplementary file 1 — Figures S1–S7 [file ACEL-23-e14202-s002.pdf]
