# Supplementary material for: Nrf2 activation by pyrroloquinoline quinone inhibits natural aging‐related intervertebral disk degeneration in mice
Source: Aging Cell. 2024 May 23;23(8):e14202. doi: 10.1111/acel.14202 (PMC11320358; doi:10.1111/acel.14202)
Supplement: Supplementary file 2 — Tables S1–S3 [file ACEL-23-e14202-s001.docx]

**Supplementary legends**

**Fig. S1.** **Body weight, serum ALT, AST and CERA levels upon PQQ supplementation in aged wild-type mice.**

(A) X-ray scans in vivo. (B) Body weight, and serum (C) ALT, (D) AST and (E) CERA levels in indicated groups of mice. One-way ANOVA with Tukey’s post-hoc test. ns: not significant.

**Fig. S2.** **DNA damage and SASP in IVD were rescued in PQQ-treated aged mice as compared to vehicle-treated control.**

(A-B) Representative images of IVD sections immunostained for 8-OHdG, IL-6 and IL-1β in (A) NP and (B) AF. (C-E) Quantification of the percentage of (C) 8-OHdG^+^ NPCs and AFCs, (D) IL-6^+^ area and (E) IL-1β^+^ area in NP and AF. One-way ANOVA with Tukey’s post-hoc test. *: p < 0.05, **: p < 0.01, ***: p < 0.001.

**Fig. S3. TUNEL assay in NP tissues of indicated groups of mice**

(A) TUNEL staining. (B) Quantification of the percentage of TUNEL^+^ cells in NP tissues. One-way ANOVA with Tukey’s post-hoc test. *: p < 0.05, ***: p < 0.001.

**Fig. S4. The effects of PQQ on cell viability and Nrf2-ARE signaling in human NPCs with short-term IL-1β stimulation.**

(A) qPCR detection of Nrf2, HO1, Gclc and Gclm in human NPCs in the presence and absence of IL-1β for 0.5, 1, 3 and 6 hours. (B) Western blot detection of whole-cell Nrf2 and nuclear Nrf2 in indicated groups. (C) Cell viability assay. One-way ANOVA with Tukey’s post-hoc test. *: p < 0.05, **: p < 0.01.

**Fig. S5. The mRNA level of Wnt5a is decreased in human NPCs with Nrf2 knockdown**

(A) qPCR detection of Wnt5a in human NPCs treated with si-control or si-Nrf2. Two-tailed Student’s t test. *: p < 0.05, **: p < 0.01, ***: p < 0.001.

**Fig. S6. Nrf2 deficiency largely blocks the inhibitory effects of PQQ supplementation on DNA damage and SASP in aged mice.**

(A) Representative images of IVD sections immunostained for 8-OHdG, IL-6 and IL-1β in (A) NP and (B) AF. (C-E) Quantification of the percentage of (C) 8-OHdG^+^ NPCs and AFCs, (D) IL-6^+^ area and (E) IL-1β^+^ area in NP and AF. One-way ANOVA with Tukey’s post-hoc test. **: p < 0.01, ***: p < 0.001. ns: not significant.

**Fig. S7. Knockdown of Nrf2 compromises the inhibitory effects of PQQ on oxidative stress and cellular senescence in vitro.**

(A) Western blot detection of Nrf2 in human NPCs treated with si-control or si-Nrf2. (B) Real-time PCR detection of *Nqo1*, *Gclc*, *Col2a1* and *Mmp13*. (C) Crystal violet staining. (D) The Quantitative analysis of (C). (E) EdU staining. (F) The percentages of EdU^+^ cells. (G) ROS levels detection using DHE staining. (H) The Quantitative analysis of ROS. (I) SA-β-gal staining. (J) The percentage of β-gal^+^ cells. One-way ANOVA with Tukey’s post-hoc test. *: p < 0.05, **: p < 0.01, ***: p < 0.001.

**Supplementary tables**

**Supplementary Table 1. Primers used for ChIP-PCR**

|  | Forward | Reverse |
| --- | --- | --- |
| Wnt5a-Site1  Wnt5a-Site2 | CTGTCAGTCAGTGGGGACAC  TACCGGAGGATAGGGTGGTG | AAGCCACAAGTTCAGCTCCA  GGTCATGCTGCCGATATGGA |

**Supplementary Table 2. Primers used for quantitative real-time PCR**

|  | Species | Forward | Reverse |
| --- | --- | --- | --- |
| Gapdh | Human | ACAACTTTGGTATCGTGGAAGG | GCCATCACGCCACAGTTTC |
| HO-1 | Human | AAGACTGCGTTCCTGCTCAAC | AAAGCCCTACAGCAACTGTCG |
| Nqo1 | Human | GAAGAGCACTGATCGTACTGGC | GGATACTGAAAGTTCGCAGGG |
| MMP13  P16  Col2a1 | Human  Human  Human | CCAGACTTCACGATGGCATTG  ATGGAGCCTTCGGCTGACT  CCAGATGACCTTCCTACGCC | GGCATCTCCTCCATAATTTGGC  GTAACTATTCGGTGCGTTGGG  TTCAGGGCAGTGTACGTGAAC |
| Gapdh | Mouse | AGGTCGGTGTGAACGGATTTG | TGTAGACCATGTAGTTGAGGTCA |
| Wnt5a | Mouse | CAACTGGCAGGACTTTCTCAA | CATCTCCGATGCCGGAACT |

**Supplementary Table 3. Indicated promoter sequences of Wnt5 cloned to pGL3-basic**

| **Wnt5a promoter-****pGL3-basic** |
| --- |
| GGATGTAAGCCTAGAGAGGACTGGAGGGAAAGTGGCCCAAGGTTTTGAGTACCTCTGGCTTTCCTAGAGACTACAAGCATCCTTAGCAAAGGAAGTCCAGTGGGGATGGCCTGAATCCATCCATCTGTCCCAGTTTACACTTCCAGCAAAAACACCAGGGGTGCAGTGATGGGAGGCTGTCAGTCAGTGGGGACACATGT**GGCTGGGTCAG**TGGAGGAACAGTCACATCCATGGAGCTGAACTTGTGGCTTATGCATGCTCTAGGGCGCGCTGCTGGGCATGGGGTAAGGCACAGGGTAGCAAACACAAACTGTCAAAGGCAAGCCAGTGGATGAAGACAACTGACTCTGGTGAGTGAGTGGAGTGTGTGTGTGTGTGTGTGTTTAATATGTGTGTGGTATGTGTGTGTGT |
| **Wnt5a promoter mutant-pGL3-basic** |
| GGATGTAAGCCTAGAGAGGACTGGAGGGAAAGTGGCCCAAGGTTTTGAGTACCTCTGGCTTTCCTAGAGACTACAAGCATCCTTAGCAAAGGAAGTCCAGTGGGGATGGCCTGAATCCATCCATCTGTCCCAGTTTACACTTCCAGCAAAAACACCAGGGGTGCAGTGATGGGAGGCTGTCAGTCAGTGGGGACACATGT***************TGGAGGAACAGTCACATCCATGGAGCTGAACTTGTGGCTTATGCATGCTCTAGGGCGCGCTGCTGGGCATGGGGTAAGGCACAGGGTAGCAAACACAAACTGTCAAAGGCAAGCCAGTGGATGAAGACAACTGACTCTGGTGAGTGAGTGGAGTGTGTGTGTGTGTGTGTGTTTAATATGTGTGTGGTATGTGTGTGTGT |
